# Lipophilicity Studies on Thiosemicarbazide Derivatives

**DOI:** 10.3390/molecules22060952

**Published:** 2017-06-08

**Authors:** Agata Paneth, Anna Hawrył, Tomasz Plech, Mirosław Hawrył, Ryszard Świeboda, Dominika Janowska, Monika Wujec, Piotr Paneth

**Affiliations:** 1 Department of Organic Chemistry, Medical University, Chodźki 4a, 20-093 Lublin, Poland; tomasz.plech@umlub.pl (T.P.); dominika.hagel@gmail.com (D.J.); monika.wujec@umlub.pl (M.W.); 2 Department of Inorganic Chemistry, Medical University, Chodźki 4a, 20-093 Lublin, Poland; anna.hawryl@umlub.pl (A.H.); mirek.hawryl@umlub.pl (M.H.); ryszard.swieboda@umlub.pl (R.Ś.); 3 Institute of Applied Radiation Chemistry, Lodz University of Technology, Żeromskiego 116, 90-924 Łódź, Poland; piotr.paneth@p.lodz.pl

**Keywords:** thiosemicarbazide derivatives, RP-HPLC, *logP*, PCA, bacterial type IIA topoisomerases

## Abstract

The lipophilicity of two series of thiosemicarbazide derivatives was assessed by the RP-HPLC method with the RP-18 chromatographic column and the methanol–water mixture as the mobile phase. Distribution coefficients *logP_HPLC_* were compared to calculated values generated by commonly used *AClogP* software and quantum chemical calculations. The reliability of the predictions was evaluated using the correlation matrix and PCA. For 4-benzoylthiosemicarbazides, a high correlation between theoretical and experimental *logP* parameters was obtained using the *XlogP3* algorithm, while for 4-aryl/(cyclohexyl)thiosemicarbazides, the *XlogP2* parameter was strongly correlated with the experimentally obtained *logP*.

## 1. Introduction

In drug discovery projects, the ability to show a relationship between compounds’ molecular structures and their pharmacokinetic parameters, in vivo efficacy, or toxicity is paramount for the design of better analogues. To aid this understanding, taking measurements of lipophilicity expressed by the logarithm of the octanol–water partition coefficient *logP* or the distribution coefficient *logD* (if ionized molecular species are present) is common practice, as these parameters are considered the most important for rational drug design and deriving a quantitative structure activity relationship (QSAR), quantitative structure retention relationship (QSRR) or a quantitative structure property relationship (QSPR) [1,2,3,4,5]. Numerous literature reports relate lipophilicity to undesirable ADMET (absorption, disposition, metabolism, excretion, and toxicity) properties, including poor solubility, poor bioavailability, high protein binding, high affinity to microsomes and hepatocytes, and in vivo toxicological effects [6,7,8,9,10,11,12]. It is therefore recommended that the lipophilicity of drug candidates be determined to eliminate molecules with unfavorable pharmacokinetic and pharmacodynamic parameters before bioassay is undertaken [13].

In this respect, there is a strong interest in developing computational methods for rapid lipophilicity screening of potential drug candidates. In recent years, numerous theoretical methods have been developed for the prediction of lipophilicity. The nature of lipophilicity is complex, however, as the outcome of inter- and intra-molecular interactions is far from being precisely encoded in the various algorithms, and the reliability of the available software depends on the chemical structure and the inherent conception of the method [14]. Consequently, for new chemotypes synthesized, computed *logP* values may be misleading for modeling their biological activity or for estimating their permeability potential [15]. Therefore, the routine application of a theoretical approach requires a comparison of the results with the data obtained using experimental methods, with particular regard to cases where intramolecular H-bonding, conformational flexibility, and/or tautomerization is possible [16].

Recently, as part of our efforts to discover new molecules that might be used to combat clinically significant infections, for the first time we have documented inhibitory properties of 1,4-disubstituted thiosemicarbazides towards bacterial DNA gyrase and topoisomerase IV [17,18,19]. Unfortunately, results of the bacterial type IIA topoisomerases inhibition study did not parallel the antibacterial activities. A possible explanation is that tested thiosemicarbazides may have varied in cell membrane permeability. We have, therefore, focused our attention on finding a computational tool that would be applicable to rapid and accurate prediction of lipophilicity for thiosemicarbazide-based compounds. In this contribution, we present results of these investigations, which allowed us to conclude that *XlogP3*, *XlogP2*, and SMD/B3LYP/def2-TZVP calculations are promising for theoretical prediction of lipophilicity for this class of compounds.

## 2. Results and Discussion

### 2.1. The Relationship between the Retention Parameter logk and the Concentration of Organic Modifier φ

The chromatographic lipophilicity parameters *logk_w_* for thiosemicarbazide derivatives **1**–**17** were obtained by the extrapolation of the retention parameter *logk* to pure water, according to Equation (1):

*logk = logk_w_ − S × φ* (1)

where *logk_w_* is the value of the retention factor of a substance in pure water, *S* is the slope of the regression curve, and *φ* is the concentration of the organic modifier.

Raw data which were used for the determination of the dependence of *logk* for different concentrations of the organic modifier are included as supplementary material (Table S1). Seven standards with known *logP* were chromatographed under the same conditions and respective data are given in Table S2.

As can be seen from results collected in Table 1, an excellent fit (*r* ≥ 0.98) of Equation (1) to the experimental data was observed in all cases [20].

### 2.2. The Calibration Equation logP vs. logk_w_

Subsequently, *logP_HPLC_* parameters for thiosemicarbazide derivatives **1**–**17** were determined, based on the correlation between the chromatographic lipophilicity index *logk_w_* and the octanol–water partition coefficient *logP_o–w_* obtained by the shake flask method for selected standards [21,22]:

*logP_o/w_ = a × logk_w_ + b* (2)

Seven standards were analyzed in the same chromatographic conditions as for compounds **1**–**17**. The *logk_w_* values of standards aniline (1.0186), benzene (2.2084), bromobenzene (3.0546), arametere (3.5502), toluene (2.8467), ethylbenzene (3.3754) and 2-hydroxyquinoline (1.3682) were obtained, based on the relationships between the retention parameters *logk*.

In order to calculate the lipophiliciy parameter *logP_o/w_* of thiosemicarbazide derivatives **1**–**17**, the linear calibration curve was prepared:

*logP_o/w_* = 1.0239 (±0.03) × *logk_w_* − 0.1541 (0.08); *n* = 7; *r* = 0.9976; *s_e_* = 0.08; *F* = 1047.8 (3)

It should be noted that this calibration curve is based on compounds selected from the OECD approved standards, all of which are structurally different from the compounds studied herein. This approach has been used earlier [23,24].

The *logk* values of **1**–**17** were substituted into Equation (3) to obtain *logP_HPLC_* parameters. The values of calculated and experimental lipophilicity parameters are presented in Table 2.

### 2.3. Theoretical Calculation clogP

**Figure 1:**
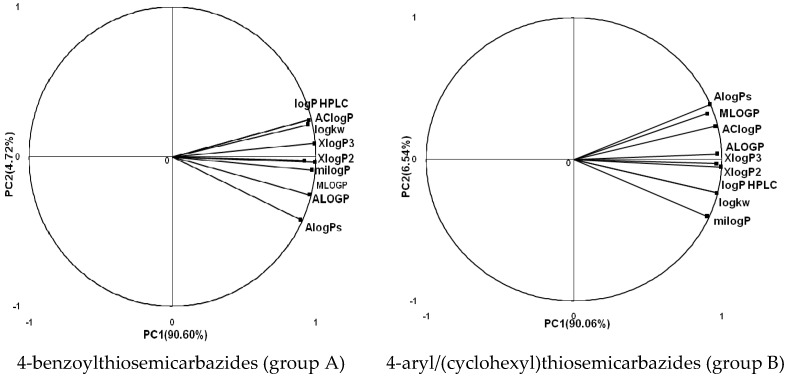
The PCA graphs for calculated and experimental lipophilicity parameters of 4-benzoylthiosemicarbazides (group A) and 4-aryl/(cyclohexyl)thiosemicarbazides (group B).

In the next step, the comparison between the experimental *logP_HPLC_* and the calculated values *logP* was carried out. For analysis, thiosemicarbazide derivatives **1**–**17** were divided into two groups; the first one (group A) included 4-benzoylothiosemicarbazides **1**–**10**, and the second one (group B) included 4-aryl and 4-cyclohexylthiosemicarbazides **11**–**17**. Based on the correlation matrices (Table 3 and Table 4) and PCA (Figure 1), it was found that for group A, the highest correlation was obtained using the *XlogP3* program, while for group B the highest correlation was obtained using the *XlogP2* program.

### 2.4. Correlation of Lipophilicity with Inhibitory Potency towards Bacterial Type IIA Topoisomerases

Lipophilicity is usually related to biological activity; the evidence for this is clearly explained in the frame of the lipid theory of Meyer and Overton [25], according to which *logP* is not only a function of the penetration and distribution of the drug, but also a function of its interaction with the molecular target. Therefore, the second part of our studies was dedicated to determining the role of lipophilicity on the inhibitory action of 1-hetaroyl-4-substituted-thiosemicarbazides against bacterial type IIA topoisomerases. Lead compounds from this series were reported to be potent and non-toxic inhibitors [17,18,19] (Table 5) and can be considered as a starting point for the development of improved antibacterial agents. Although a correlation of their inhibitory potency with hydrophobic/hydrophilic balance was suggested to exist, no such trend could be deduced from the comparison of their calculated *logP* values. A possible explanation for the lack of such correlation could be the use of unreliable research tools used for lipophilicity prediction. We therefore repeated *logP* calculations using the *XlogP3* and *XlogP2* programs, as these were recognized to be reliable tools for the prediction of lipophilicity for this class of compounds.

A set of seventeen 1-hetaroyl-4-substituted-thiosemicarbazides **1**, **2**, **18**–**32**, previously tested as *S. aureus* DNA gyrase and topoisomerase IV (topo IV) inhibitors, was used for model generation. The analysis of calculated *logP* values for the compounds, however, leads to ambiguous conclusions. According to results collected in Table 5, within series of 4-benzoylthiosemicarbazides **1**, **2**, **18**–**20**, the best inhibitory potency against DNA gyrase was found for **1** (IC_50_ 14.59 μM), with *XlogP3* = 2.59. The replacement of furan moiety in **1** with indole **18** or imidazole **20** leads to inactive compounds with a lipophilicity respectively higher (*XlogP3* of 3.59) or lower (*XlogP3* of 2.00) than that of **1,** whereas, for instance, *XlogP3* for inhibitors with pyrrole **2** and thiophene **19** substitution lie at 2.25 and 3.14, respectively. Again, **18** (*XlogP3* of 3.59), **2** (*XlogP3* of 2.25) and **20** (*XlogP3* of 2.00) were found to have anti-topo IV activity, while other compounds of similar lipophilicity, **1** (*XlogP3* of 2.59) and **19** (*XlogP3* of 3.14), were inactive. No valid correlation between inhibitory activity of 4-arylthiosemicarbazides **21**–**32** against topo IV and their logP values expressed as *XlogP2* was observed. For instance, within series **21**–**32**, the best anti-topo IV activity was found for **21** (IC_50_ 14 μM, *XlogP2* of 2.82). The replacement of the 4-nitrophenyl group in **21** by *o*-fluoro-, *p*-fluorophenyl or 2,4-difluorophenyl groups, as in **22** (*XlogP2* of 3.09), **23** (*XlogP2* of 3.09) and **24** (*XlogP2* of 3.25), leads to compounds with lipophilicity similar to **21** that, however, show only weak inhibitory potency. To conclude, although the most lipophilic of 4-benzoylthiosemicarbazides (**18**) inhibited topo IV most effectively, no linear relationship between the inhibitory potency of series 4-benzoylthiosemicarbazides against DNA gyrase and their lipophilicity was observed. No relationship between the inhibitory potency of series 4-arylthiosemicarbazides against bacterial topoisomerases and their lipophilicity was found. Virtually the same conclusion was reached by Tanitame et al. [26] in studies on the inhibitory activity of 5-vinylpyrazoles against bacterial DNA gyrase. The idea behind their concept was to design more potent 5-vinylpyrazoles by decreasing the lipophilicity of the parent compounds, while keeping the van der Waals interaction with the lipophilic area around Ile94 of DNA gyrase. Among the six analogs obtained, two compounds showed less potent inhibitory activity, while the bioactivity of others was similar or only modestly better. Finally, no linear relationship between the inhibitory potency of 5-vinylpyrazoles against DNA gyrase and their lipophilicity could be deduced.

For comparison, we have tested quantum-chemical calculations of *logP*. From a thermodynamic point of view, the equilibrium constant *K* (at a given temperature and standard concentrations) is given by Equation:

∆*G = −RTlnK* (4)

where *G* is Gibbs free energy, *R* is the universal gas constant and *T* is the absolute temperature.

**Figure 2:**
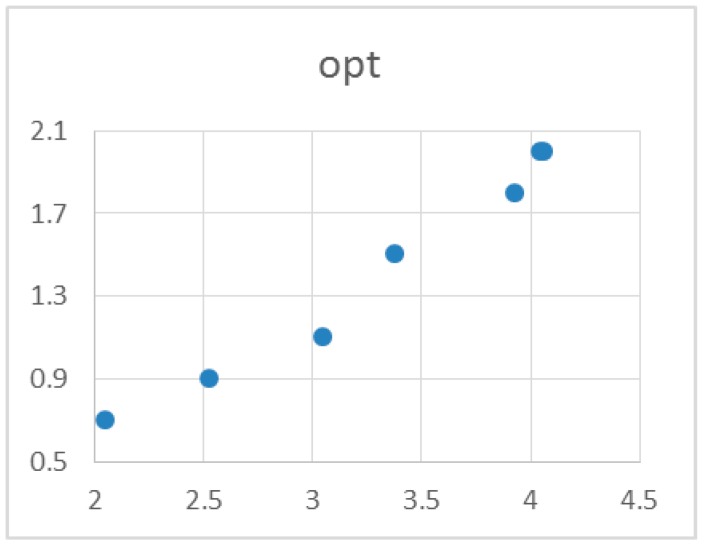
Correlation of experimental *logP* with theoretical results obtained at the SMD/B3LYP/def2-TZVP level.

It thus should be possible to evaluate *logK_o–w_* values by computing Gibbs free energies of a given molecule in aqueous and 1-octanol solutions. Expanding recent reports [27], we have tested three continuum solvent models (IEFPCM, CPCM, SMD) with the DFT B3LYP functional expressed in several basis sets (6-31+G(p,d), 6-311++G(d,p), aug-cc-pVDZ, def2-DZVP, def2-TZVP). Initially, RM1 optimized structures were reoptimized in the gas phase at the given theory level, and three types of calculations were then performed. Firstly, energies for the gas phase structures with inclusion of the solvent model were calculated. Secondly, reoptimization with the solvent model included was performed for both liquid phases. Finally, frequency calculations for the structures optimized with solvent models were carried out in order to calculate zero point energies (ZPEs) and thermal corrections to Gibbs free energies. We have found double-zeta basis sets to be inadequate, as they produced inverse solution stabilities compared with the experimental results. Among tested methods, only SMD/B3LYP/def-TZVP turned out to be promising in terms of speed of calculations and agreement of the results with the experimental data, with equation *logP* = 1.46·*logK_o–w_* + 1.21 (Figure 2) describing the correlation between the theoretical (*K_o–w_* is the calculated equilibrium constant) and experimental results. However, slight deviation from linearity, the slope differing from unity, and the intercept differing from zero indicate that calibration is necessary for each class of studied compounds.

## 3. Materials and Methods

### 3.1. Chromatographic Analysis

The chromatographic analysis was performed using a liquid chromatograph equipped with an Elite LaChrom L-2130 gradient pump (Hitachi-Merck, Darmstadt, Germany), SPD-10AVP UVVIS detector (Shimadzu, Kyoto, Japan), and Rheodyne 7725i valve with a 20 μL loop. Methanolic solutions (5 μL, 0.1%) of selected samples were applied to the chromatographic column (RP-18 Waters Symmetry, 15 cm length, 4.6 mm i.d., 5 μm particle size) by use of an autosampler Hitachi L-2200 (LaChrom Elite, Hitachi-Merck, Darmstadt, Germany). The mobile phase, consisting of a methanol and water mixture, was degassed by use of the built-in membrane degasser. The analysis was performed with a flow rate of 1.0 mL/min in isocratic mode using various concentrations of organic modifier in binary polar mobile phases; percentages of methanol in water were 45–75% (*v*/*v* %), and changed by 5% per step. Chromatograms were detected at 254 nm and the temperature of the column was 25 °C. All experiments were repeated in triplicate and the final results were taken to be the arithmetic means. Dead time was measured by use of uracil (Calbiochem. Merck, Darmstadt, Germany). All experiments were performed at ambient temperature.

### 3.2. Standard Solutes

According to OECD guidelines [28], seven standards with known *logP_o/w_* values—aniline (0.9), benzene (2.1), bromobenzene (3.0), naphtalene (3.6), toluene (2.7), ethylbenzene (3.2) and 2-hydroxyquinoline (1.26)—were selected to create the correlation between the known *logP_o/w_* and the experimental chromatographic lipophilicity parameter *logk_W_*. The obtained calibration curve was used to calculate the *logP_o/w_* of compounds **1**–**17** under the same chromatographic conditions.

### 3.3. Statistical Analysis

All regression analyses were performed using statistical software (Statistica version 9.0 for Windows).

### 3.4. LogP Calculations

The theoretical partition coefficient values were calculated using the available ALOGPS 2.1 software program [29,30]. The calculations were based on the analysis of algorithm topology of the whole molecules of studied compounds (*AClogP*, *AlogPs* and *MLOGP*) and the analysis of individual atoms (*ALOGP*, *XlogP2* and *XlogP3*).

### 3.5. Quantum-Chemical Calculations

Initial models were prepared and optimized by RM1 semiempirical parametrization [31] using Hyperchem (version 8.0.3, HyperCube Inc., Gainsville, FL, USA). They were subsequently reoptimized in the gas phase, IEFPCM [32], CPCM [33], and SMD [34] continuum models of the aqueous solution and 1-octanol at the DFT level using the B3LYP functional [35,36]. The performance of four basis sets (6-31+G(p,d), 6-311++G(d,p) [37,38,39], aug-cc-pVDZ [40,41,42,43], def2-DZVP [44]) have been tested. Finally, Gibbs free energies of the studied compounds were obtained from vibrational analysis. All these calculations were performed using a Gaussian computational package [45].

## 4. Conclusions

Distribution coefficients *logP* for two series of thiosemicarbazide derivatives were experimentally determined by the RP-HPLC method and correlated with those obtained using *AClogP* software and quantum-chemical calculations. For 4-benzoylthiosemicarbazides, the best results were achieved using the *XlogP3* algorithm, while for derivatives of compounds 4-aryl/(cyclohexyl)thiosemicarbazides, *XlogP2* parameters were strongly correlated with experimentally obtained *logP*. Among tested theory levels, only SMD/B3LYP/def2-TZVP results seem to be useful in theoretical prediction of lipophilicity for this class of compounds, although calibration is necessary.

## Figures and Tables

**Table 1:** Parameters of Equation (1). Obtained *F* values were higher than *F*-critical in each case.

Compound		*logk_w_*	−*S*	*r*	*n*	*F*	SD of Estimation
**1**	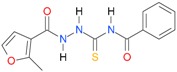	2.9992	4.3720	0.9986	7	1796.1	0.027
**2**	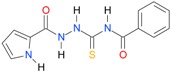	2.5105	4.1416	0.9983	7	1487.0	0.028
**3**	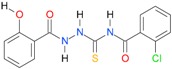	4.2230	6.1166	0.9983	7	1440.4	0.043
**4**	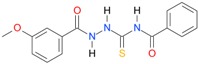	3.3130	4.8205	0.9984	7	1598.1	0.032
**5**	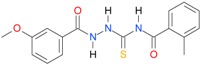	3.4487	4.8562	0.9987	7	1864.2	0.030
**6**	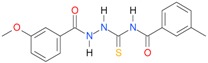	3.7864	5.1137	0.9976	7	1049.6	0.042
**7**	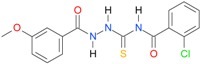	3.8283	5.6579	0.9934	7	372.8	0.078
**8**	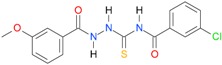	3.7422	5.0742	0.9972	7	901.4	0.045
**9**	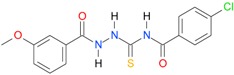	3.9422	5.3417	0.9974	7	975.2	0.045
**10**	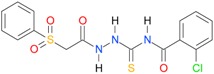	3.2498	5.7291	0.9838	7	150.3	0.124
**11**	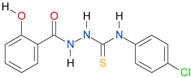	3.3324	4.8778	0.9983	7	2138.6	0.028
**12**	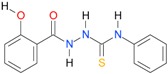	2.4813	4.1962	0.9992	7	3260.5	0.019
**13**	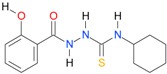	3.2386	4.7076	0.9987	7	1871.6	0.029
**14**	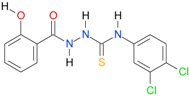	4.1453	5.6538	0.9980	7	1251.7	0.042
**15**	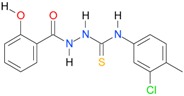	3.9451	5.5432	0.9964	7	690.2	0.056
**16**	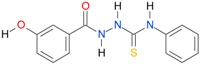	2.0687	4.1968	0.9993	7	3508.6	0.019
**17**	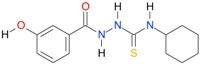	2.7583	4.3297	0.9993	7	3507.0	0.019

**Table 2:** The values of calculated and experimental lipophilicity parameters of thiosemicarbazides **1**–**17**.

Compound	*AlogP_s_*	*AclogP*	*milogP*	*AlogP*	*MlogP*	*XlogP2*	*XlogP3*	*logk_w_*	*logP_HPLC_*
**1**	0.90	1.70	1.28	1.80	1.33	1.62	2.59	2.9992	3.0469
**2**	1.66	1.06	1.27	1.94	1.06	1.59	2.25	2.5105	2.5254
**3**	2.86	2.59	2.23	2.95	3.20	3.43	3.95	4.2300	4.3606
**4**	2.36	2.17	2.15	2.53	2.42	2.70	3.10	3.3130	3.3819
**5**	2.41	2.49	2.55	3.02	2.66	3.14	3.46	3.4487	3.5267
**6**	2.41	2.49	2.57	3.02	2.66	3.14	3.46	3.7864	3.8871
**7**	3.00	2.79	2.78	3.20	2.93	3.32	3.73	3.8283	3.9318
**8**	2.93	2.79	2.80	3.20	2.93	3.32	3.73	3.7422	3.8399
**9**	2.88	2.79	2.83	3.20	2.93	3.32	3.73	3.9422	4.0534
**10**	2.62	1.59	1.53	2.81	2.57	2.43	3.06	3.2498	3.3144
**11**	2.82	2.66	3.31	3.08	3.39	3.41	3.52	3.3324	3.4026
**12**	1.95	2.05	2.63	2.41	2.86	2.78	3.21	2.4813	2.4942
**13**	1.72	2.05	3.48	2.69	2.44	3.05	3.14	3.2386	3.3025
**14**	3.32	3.27	3.91	3.74	3.64	4.03	4.15	4.1453	4.2702
**15**	3.02	2.97	3.69	3.56	3.64	3.63	3.88	3.9451	4.0565
**16**	1.99	2.05	1.64	2.41	2.35	2.36	2.34	2.0687	2.0538
**17**	1.74	2.05	2.48	2.69	1.93	2.62	2.59	2.7583	2.7898

**Table 3:** The correlation matrix for *logP_calc_* vs. *logP_HPLC_* (group A: 4-benzoylthiosemicarbazides).

	*AlogP_s_*	*AclogP*	*milogP*	*AlogP*	*MlogP*	*XlogP2*	*XlogP3*	*logk_w_*	*logP_HPLC_*
*AlogP_s_*	1.0000	0.7241	0.7833	0.9335	0.9132	0.8906	0.8518	0.7676	0.7676
*AclogP*		1.0000	0.9500	0.8541	0.8641	0.9348	0.9403	0.9052	0.9052
*milogP*			1.0000	0.8993	0.8226	0.9282	0.8755	0.8013	0.8013
*AlogP*				1.0000	0.9425	0.9529	0.9177	0.8361	0.8361
*MlogP*					1.0000	0.9632	0.9667	0.9251	0.9251
*XlogP2*						1.0000	0.9776	0.9265	0.9265
*XlogP3*							1.0000	0.9716	0.9716
*logk_w_*								1.0000	1.0000
*logP_HPLC_*								1.0000	1.0000

**Table 4:** The correlation matrix for *logP_calc_* vs. *logP_HPLC_* (group B: 4-aryl/(cyclohexyl)thiosemicarbazides).

	AlogP_s_	AclogP	milogP	AlogP	MlogP	XlogP2	XlogP3	logk_w_	logP_HPLC_
AlogP_s_	1.0000	0.9809	0.6650	0.9168	0.9309	0.8909	0.8564	0.8014	0.8014
AclogP		1.0000	0.7475	0.9725	0.8927	0.9373	0.8881	0.8839	0.8839
milogP			1.0000	0.8199	0.7237	0.9251	0.9135	0.9520	0.9521
AlogP				1.0000	0.8142	0.9450	0.8769	0.9510	0.9510
MlogP					1.0000	0.8805	0.9201	0.7670	0.7670
XlogP2						1.0000	0.9712	0.9650	0.9650
XlogP3							1.0000	0.9136	0.9136
logk_w_								1.0000	1.0000
logP_HPLC_								1.0000	1.0000

**Table 5:** Structures and inhibitory potency of thiosemicarbazides **18**–**34** towards bacterial topoisomerases.

Compound		*XlogP3*	*XlogP2*	Inhibitory Potency IC_50_ [μM]	
				DNA Gyrase	Topo IV
**1** [16]	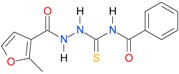	2.59	n.d. *	14.59	n.a. **
**2** [16]	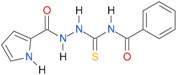	2.25	n.d.	93.30	41.04
**18** [17]	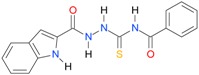	3.59	n.d.	n.d.	14
**19** [16]	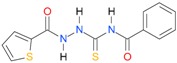	3.14	n.d.	83.63	n.a.
**20** [17]	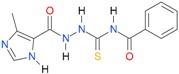	2.00	n.d.	n.a.	90.00
**21** [18]	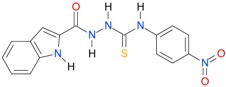	n.d.	2.82	n.a.	14.00
**22** [16]	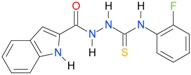	n.d.	3.09	n.a.	63.47
**23** [16]	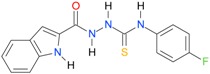	n.d.	3.09	127.68	267.04
**24** [18]	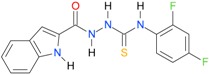	n.d.	3.25	n.a.	295.00
**25** [16]	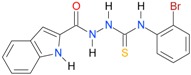	n.d.	3.73	n.a.	n.a.
**26** [18]	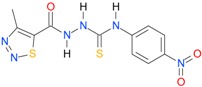	n.d.	2.25	n.a.	n.a.
**27** [16]	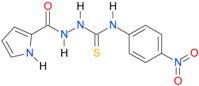	n.d.	1.46	64.21	n.a.
**28** [18]	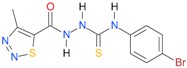	n.d.	3.16	n.a.	403.00
**29** [18]	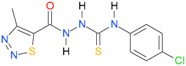	n.d.	2.98	n.a.	n.a.
**30** [18]	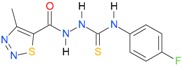	n.d.	2.52	n.a.	n.a.
**31** [18]	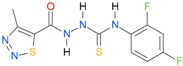	n.d.	2.68	n.a.	n.a.
**32** [18]	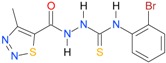	n.d.	3.16	n.a.	n.a.

* n.d.—not determined, ** n.a.—no activity.

## Supplementary Materials

Supplementary materials are available online. Table S1: The values of the retention time (**t_r_**) and *logk* for different concentrations of methanol in water (ranging from 45% to 75%) obtained on RP-18 column; Table S2: Statistics and parameters of Equation (1) obtained for standards with known *logP*.
