# Supplementary material for: Lipophilicity Studies on Thiosemicarbazide Derivatives
Source: Molecules. 2017 Jun 8;22(6):952. doi: 10.3390/molecules22060952 (PMC6152747; doi:10.3390/molecules22060952)
Supplement: Supplementary file 1 [file molecules-22-00952-s001.pdf]

Supplementary Materials: Lipophilicity studies on thiosemicarbazide derivatives

Agata Paneth [1,*], Anna Hawrył [2], Tomasz Plech [1], Mirosław Hawrył [2], Ryszard Świeboda [2], Dominika Janowska [1], Monika Wujec [1], Piotr Paneth[3]

Table S1. The values of the retention time (**t**r) and logk for different concentrations of methanol in water (ranging from 45% to 75%) obtained on RP-18 column.

Compound	45% MeOH		50% MeOH		55% MeOH		60% MeOH		65% MeOH		70% MeOH		75% MeOH	
	**t_r**	**logk**	**t_r**	**logk**	**t_r**	**logk**	**t_r**	**logk**	**t_r**	**logk**	**t_r**	**logk**	**t_r**	**logk**
Aniline	3.287	0.061674	2.840	-0.06557	2.500	-0.19573	2.260	-0.31874	2.087	-0.43565	1.960	-0.54735	1.860	-0.66139
Benzene	15.433	0.959363	11.580	0.818457	8.460	0.657082	6.567	0.518591	5.187	0.379642	4.160	0.236612	3.453	0.100817
Bromobenzene	44.687	1.451242	29.447	1.262076	18.653	1.049817	12.873	0.871004	9.107	0.69583	6.587	0.520311	4.980	0.354358
2-hydroxyquinoline	3.987	0.207096	3.233	0.04814	2.647	-0.13462	2.313	-0.28842	2.107	-0.42041	1.967	-0.54039	1.860	-0.66139
Naphtalene	75.833	1.687185	47.093	1.474802	27.840	1.236331	18.253	1.039553	12.293	0.848215	6.520	0.514523	6.173	0.48324
Toluene	35.047	1.341465	24.233	1.172302	16.133	0.980692	11.547	0.817029	7.040	0.557549	6.233	0.488813	4.813	0.332829
Ethylbenzene	73.160	1.671274	47.067	1.474554	28.813	1.252101	19.260	1.064943	13.053	0.87784	8.993	0.689249	6.487	0.511643
Uracile	1.527		1.513		1.487		1.487		1.480		1.480		1.473	
1	15.773	0.969854	9.540	0.719956	5.500	0.41528	3.900	0.191459	2.940	-0.0337	2.400	-0.24282	2.053	-0.46285
2	22.320	1.134078	14.273	0.921535	8.927	0.685393	6.187	0.484547	3.607	0.134224	2.873	-0.05479	2.527	-0.18384
10	19.320	1.06641	12.507	0.856763	3.740	0.161142	2.820	-0.07224	2.380	-0.25289	1.953	-0.55443	1.780	-0.78072
11	16.773	0.999317	11.200	0.801722	3.880	0.187783	3.000	-0.01564	1.987	-0.52108	1.767	-0.80363	1.660	-1.05999
12	15.147	0.950338	9.260	0.704509	5.447	0.409447	3.867	0.185377	2.960	-0.02759	2.407	-0.23936	2.073	-0.44665
13	19.007	1.058702	11.807	0.828154	6.980	0.552796	4.953	0.350948	3.707	0.154617	2.887	-0.0503	2.360	-0.26319
14	11.840	0.829546	7.453	0.588923	4.613	0.305557	3.413	0.091703	2.327	-0.28075	2.240	-0.33075	1.960	-0.54735

15	8.420	0.654569	5.540	0.41963	3.320	0.069741	2.687	-0.11938	2.320	-0.28457	2.073	-0.44665	1.927	-0.58178
16	8.460	0.657082	5.840	0.45094	3.927	0.196372	3.107	0.014818	2.553	-0.17269	2.213	-0.34751	1.893	-0.62036
19	26.233	1.208963	15.467	0.960424	7.807	0.614121	5.627	0.428945	4.040	0.216353	2.740	-0.09998	2.320	-0.28457
23	47.246	1.476258	25.793	1.201159	12.740	0.865883	7.147	0.565897	3.820	0.176565	2.873	-0.05479	2.273	-0.3111
A1	24.093	1.169616	14.287	0.922012	7.753	0.61037	5.400	0.404208	3.887	0.189073	2.807	-0.07663	2.340	-0.27375
A2	14.233	0.92017	10.147	0.751668	6.373	0.501544	4.873	0.340687	2.927	-0.03771	2.533	-0.18124	2.313	-0.28842
A3	32.113	1.301684	17.613	1.022609	9.860	0.736962	6.460	0.509272	4.467	0.284508	3.300	0.06487	2.547	-0.17524
A4	52.847	1.526448	28.880	1.253166	14.147	0.91722	8.820	0.679067	5.780	0.444856	4.060	0.219796	3.007	-0.01358
A5	39.060	1.390574	15.287	0.954779	8.107	0.634387	5.327	0.395945	3.840	0.180337	2.540	-0.17823	2.220	-0.34311
A6	46.367	1.467827	24.720	1.181518	15.267	0.954148	9.293	0.706358	6.313	0.496134	3.513	0.11414	2.933	-0.03585
A7	56.760	1.55836	31.087	1.286865	15.640	0.96578	9.547	0.720335	6.427	0.506357	3.627	0.13838	3.000	-0.01564
S4	17.487	1.019194	8.533	0.661631	6.653	0.52594	3.640	0.14106	2.573	-0.16431	2.180	-0.36893	2.007	-0.5026
S5	9.680	0.727478	6.470	0.510152	3.093	0.010953	2.187	-0.3643	2.007	-0.5026	1.780	-0.78072	1.713	-0.91433
S6	55.413	1.547637	31.493	1.29279	18.253	1.039553	10.733	0.780232	7.173	0.567902	5.033	0.360973	2.003	-0.50623
S7	36.933	1.365238	16.153	0.981287	8.520	0.660824	4.213	0.245267	3.333	0.072879	2.333	-0.2775	2.113	-0.41594
610	23.020	1.148458	13.920	0.909337	8.440	0.655828	4.993	0.35599	3.707	0.154617	2.773	-0.08832	2.300	-0.29566
612	7.613	0.600493	5.320	0.395144	3.827	0.177889	2.827	-0.0699	2.373	-0.25647	2.060	-0.45711	1.873	-0.64476

616	22.567	1.139207	13.587	0.897508	8.320	0.648223	5.153	0.375589	3.680	0.149205	2.873	-0.05479	2.373	-0.25647
618	64.793	1.617331	34.787	1.338083	18.767	1.052698	8.913	0.68457	5.800	0.446894	3.813	0.175237	2.907	-0.04396
624	53.087	1.528474	23.020	1.148458	12.853	0.870238	6.987	0.553354	4.733	0.322124	3.300	0.06487	2.600	-0.15324
662	3.727	0.158584	2.980	-0.02157	2.433	-0.22671	2.067	-0.45145	1.873	-0.64476	1.740	-0.85546	1.647	-1.10466
666	11.800	0.827858	7.607	0.600065	5.093	0.368342	3.607	0.134224	2.820	-0.07224	2.353	-0.26686	2.047	-0.46784
718	27.580	1.232019	17.467	1.018649	10.660	0.776774	6.933	0.549037	4.947	0.350187	3.740	0.161142	2.960	-0.02759

Table S2. Statistics and parameters of Eq. 1 obtained for standards with known logP

	Compound	$logk_w$	$-S$	r	n	F	SD of estimation
**1**	Aniline	1.1365	2.4091	0.9996	7	5569.4	0.009
**2**	Benzene	2.2458	2.8691	0.9998	7	10652.0	0.007
**3**	Bromobenzene	3.0842	3.6630	0.9993	7	3586.4	0.016
**4**	2-hydroxyquinoline	1.4879	2.9059	0.9967	7	761.8	0.028
**5**	Naphtalene	3.5779	4.2289	0.9931	7	358.5	0.059
**6**	Toluene	2.8770	3.4400	0.9946	7	456.9	0.043
**7**	Ethylbenzene	3.4018	3.8740	0.9995	7	5028.0	0.145
